# Supplementary material for: Genetic Characteristics of Wuxiang Virus in Shanxi Province, China
Source: Viruses. 2024 Jan 10;16(1):103. doi: 10.3390/v16010103 (PMC10818450; doi:10.3390/v16010103)
Supplement: Supplementary file 1 [file viruses-16-00103-s001.zip › Figure S1.pdf]

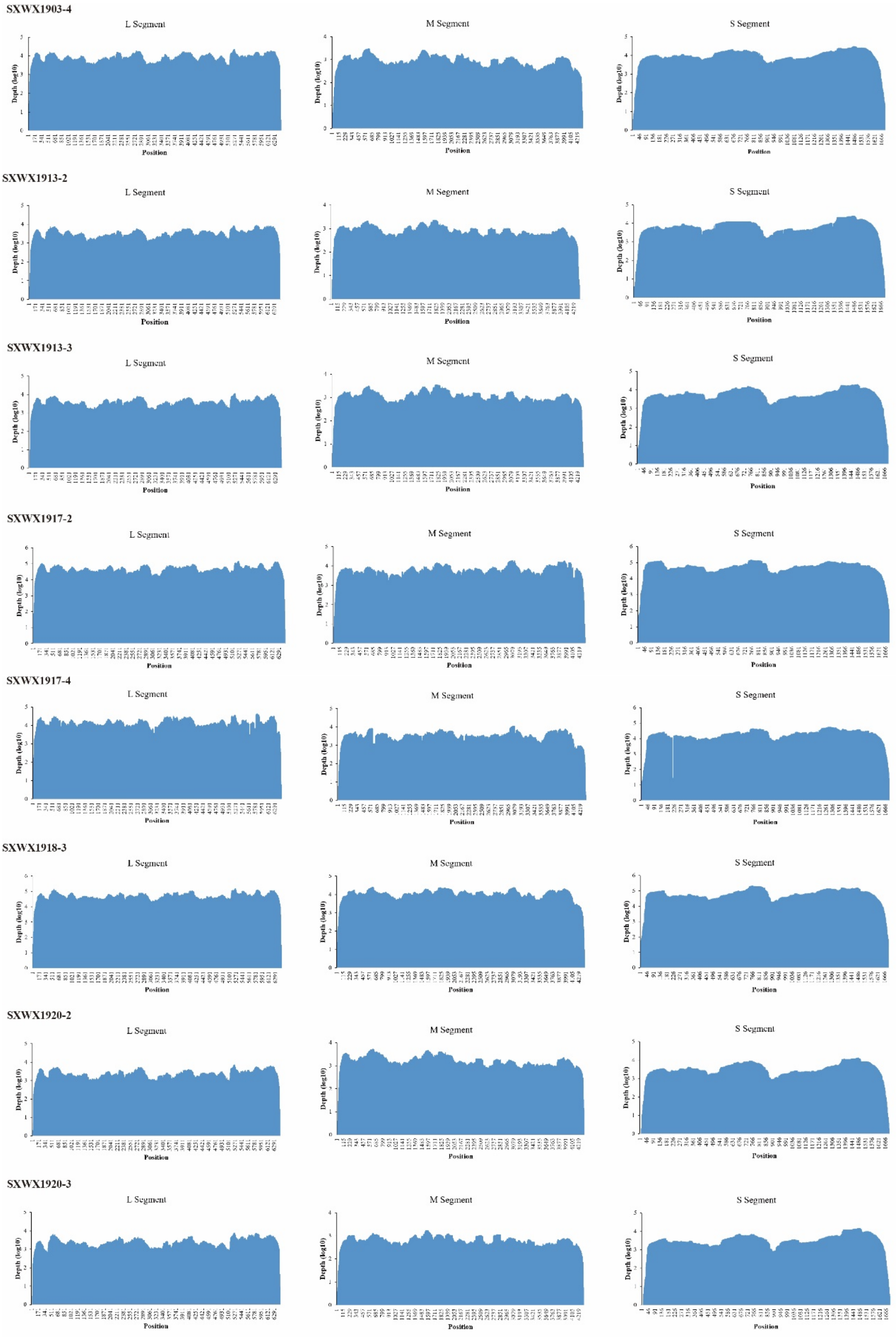

Figure S1. Continue.

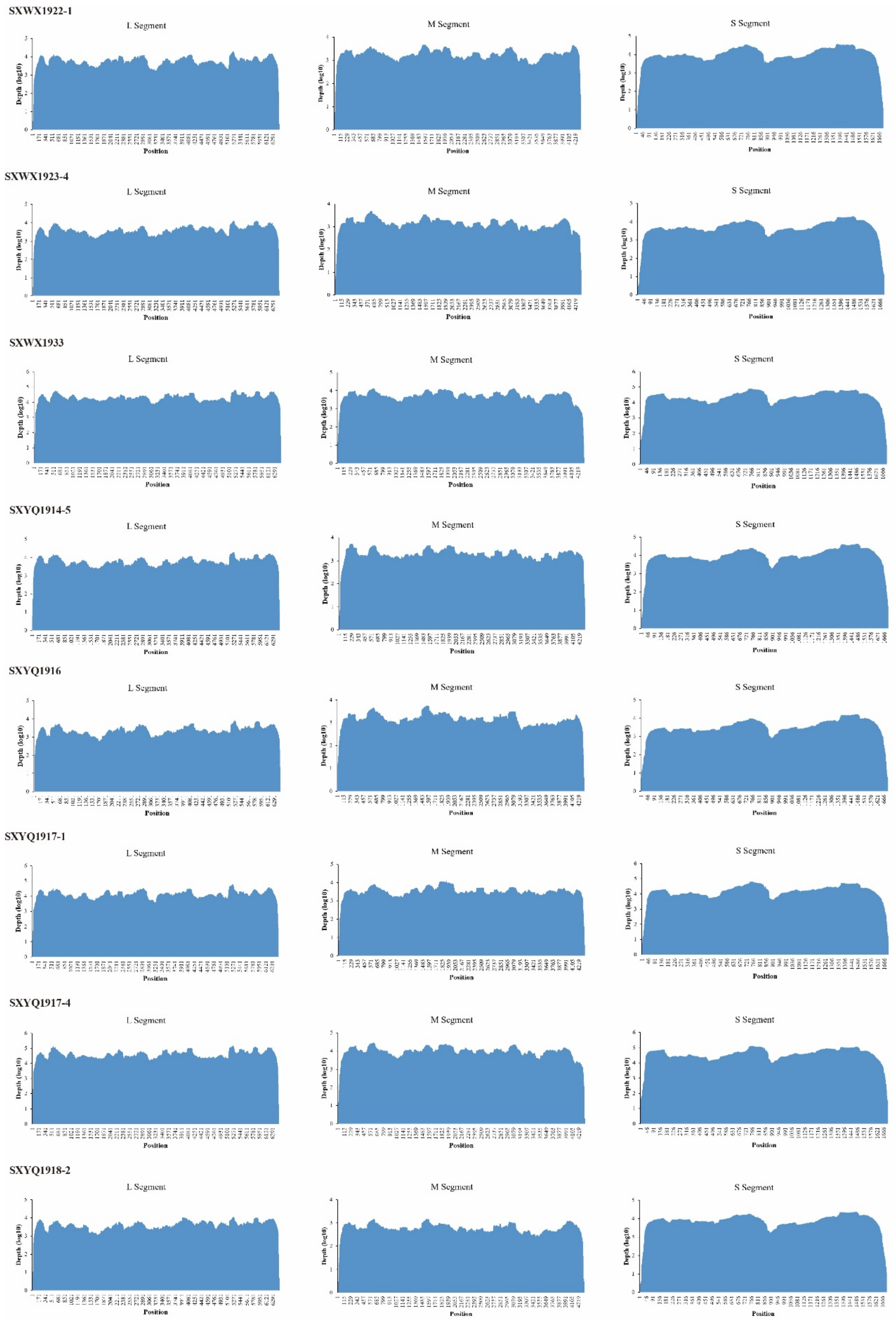

Figure S1. Continue.

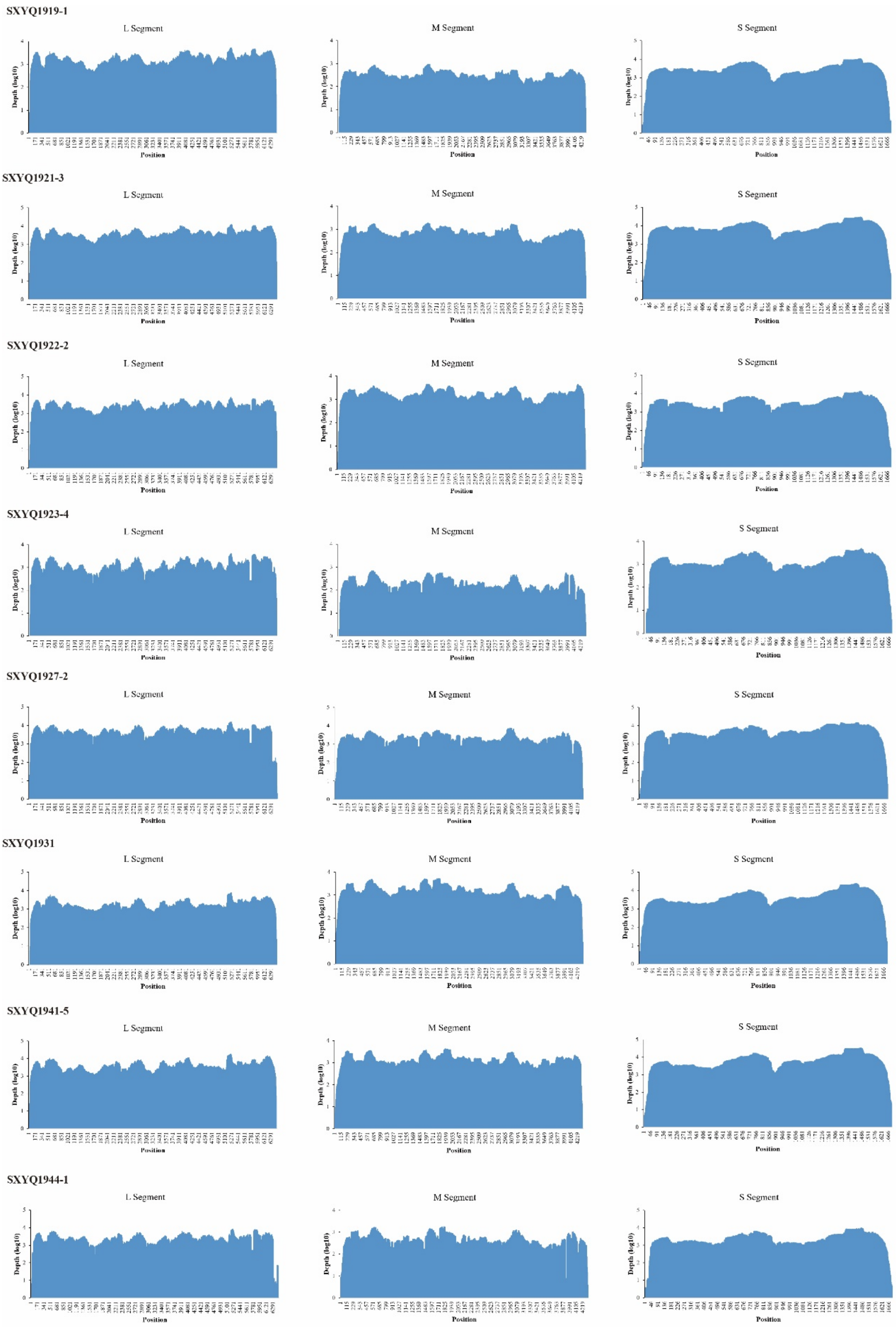

Figure S1. Continue.

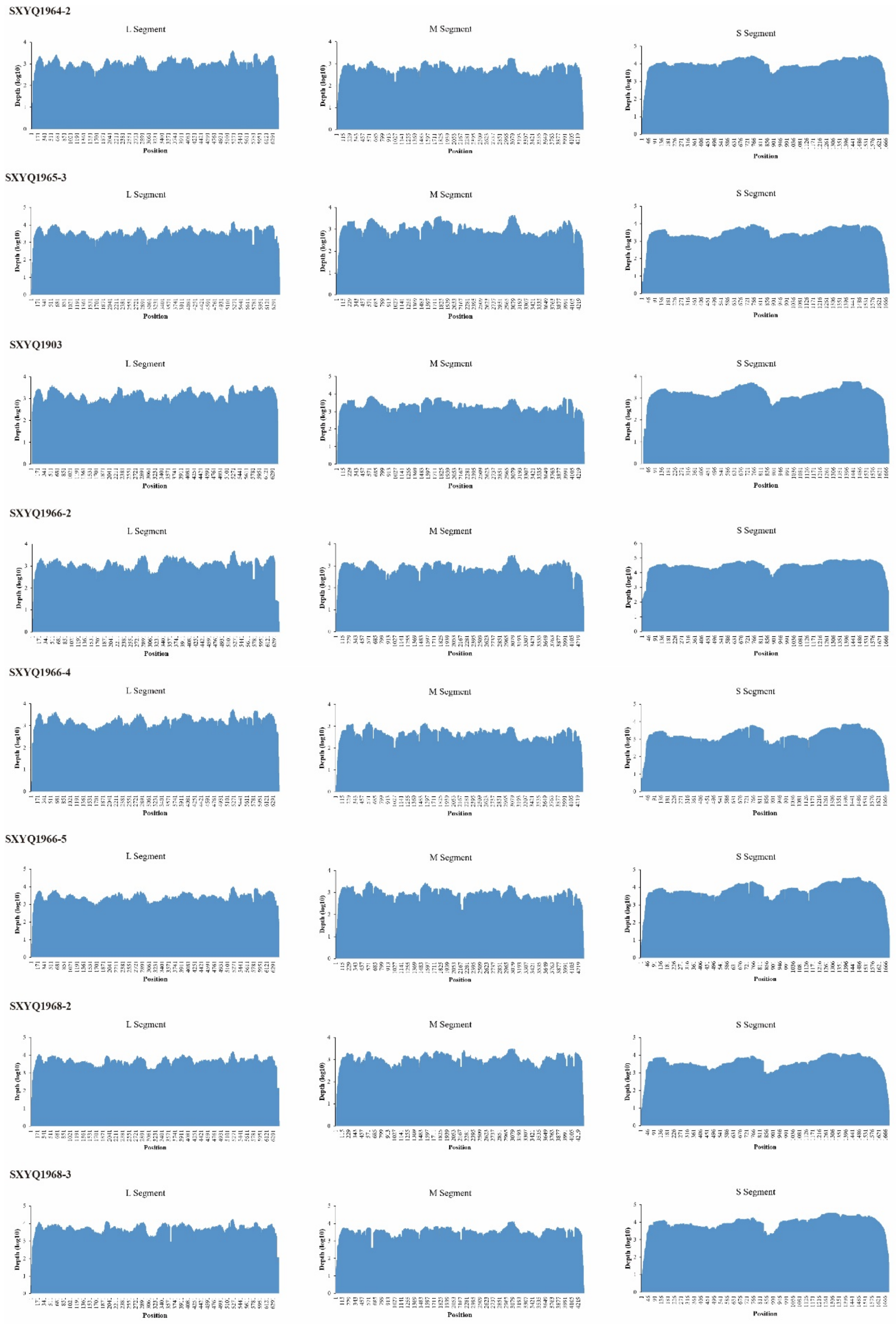

Figure S1. Continue.

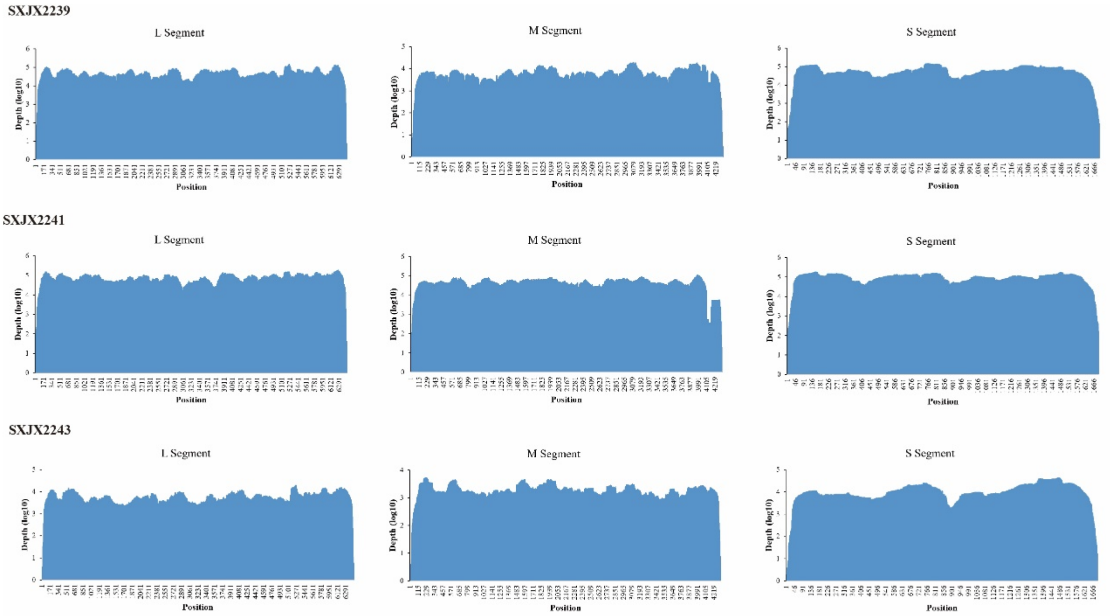

Figure S1. Coverage depth of the downstream data mapping results from the WUXV strains sequenced in this study.
