# Supplementary material for: Genetic Characteristics of Wuxiang Virus in Shanxi Province, China
Source: Viruses. 2024 Jan 10;16(1):103. doi: 10.3390/v16010103 (PMC10818450; doi:10.3390/v16010103)
Supplement: Supplementary file 1 [file viruses-16-00103-s001.zip › Table S1.pdf]

Table S1. Background information of WUXV strains used in this study.

No.	Strain	Date	Location	Host	Accession		
					L segment	M segment	S segment
1	SXJX2239	2022.07.29	Jiexiu City, Shanxi Province, China	*Phlebotomus chinensis*	C_AA050123*	C_AA050148*	C_AA050163*
2	SXJX2241	2022.07.29	Jiexiu City, Shanxi Province, China	*Phlebotomus chinensis*	C_AA050134*	C_AA050149*	C_AA050164*
3	SXJX2243	2022.07.29	Jiexiu City, Shanxi Province, China	*Phlebotomus chinensis*	C_AA050145*	C_AA050150*	C_AA050165*
4	SXWX1903-4	2019.06.14	Wuxiang County, Shanxi Province, China	*Phlebotomus chinensis*	C_AA050156*	C_AA050151*	C_AA050166*
5	SXWX1913-2	2019.06.14	Wuxiang County, Shanxi Province, China	*Phlebotomus chinensis*	C_AA050167*	C_AA050152*	C_AA050168*
6	SXWX1913-3	2019.06.14	Wuxiang County, Shanxi Province, China	*Phlebotomus chinensis*	C_AA050178*	C_AA050153*	C_AA050169*
7	SXWX1917-1	2019.06.26	Wuxiang County, Shanxi Province, China	*Phlebotomus chinensis*	C_AA050179*	C_AA050154*	C_AA050170*
8	SXWX1917-4	2019.06.26	Wuxiang County, Shanxi Province, China	*Phlebotomus chinensis*	C_AA050180*	C_AA050155*	C_AA050171*
9	SXWX1918-3	2019.06.26	Wuxiang County, Shanxi Province, China	*Phlebotomus chinensis*	C_AA050181*	C_AA050157*	C_AA050172*
10	SXWX1920-2	2019.06.26	Wuxiang County, Shanxi Province, China	*Phlebotomus chinensis*	C_AA050124*	C_AA050158*	C_AA050173*
11	SXWX1920-3	2019.06.26	Wuxiang County, Shanxi Province, China	*Phlebotomus chinensis*	C_AA050125*	C_AA050159*	C_AA050174*
12	SXWX1922-1	2019.06.26	Wuxiang County, Shanxi Province, China	*Phlebotomus chinensis*	C_AA050126*	C_AA050160*	C_AA050175*
13	SXWX1923-4	2019.06.26	Wuxiang County, Shanxi Province, China	*Phlebotomus chinensis*	C_AA050127*	C_AA050161*	C_AA050176*
14	SXWX1933	2019.07.19	Wuxiang County, Shanxi Province, China	*Phlebotomus chinensis*	C_AA050128*	C_AA050162*	C_AA050177*
15	SXWX1813-2	2018.06./	Wuxiang County, Shanxi Province, China	*Phlebotomus chinensis*	MN454526	MN454527	MN454528
16	SXYQ1903	2019.06.11	Yangquan City, Shanxi Province, China	*Phlebotomus chinensis*	C_AA050129*	MW805211	MW805193
17	SXYQ1916	2019.06.11	Yangquan City, Shanxi Province, China	*Phlebotomus chinensis*	C_AA050130*	MW192754	MW192750
18	SXYQ1918-2	2019.06.11	Yangquan City, Shanxi Province, China	*Phlebotomus chinensis*	C_AA050131*	MW805213	MW805195
19	SXYQ1919-1	2019.06.11	Yangquan City, Shanxi Province, China	*Phlebotomus chinensis*	C_AA050132*	MW805214	MW805197
20	SXYQ1921-3	2019.06.11	Yangquan City, Shanxi Province, China	*Phlebotomus chinensis*	C_AA050133*	MW805216	MW805198
21	SXYQ1931	2019.06.11	Yangquan City, Shanxi Province, China	*Phlebotomus chinensis*	C_AA050138*	MW192757	MW192753
22	SXYQ1941-5	2019.06.11	Yangquan City, Shanxi Province, China	*Phlebotomus chinensis*	C_AA050139*	MW805219	MW805201
23	SXYQ1922-2	2019.06.11	Yangquan City, Shanxi Province, China	*Phlebotomus chinensis*	C_AA050135*	MW805217	MW805199

24	SXYQ1923-4	2019.06.11	Yangquan City, Shanxi Province, China	*Phlebotomus chinensis*	C_AA050136*	MW805218	MW805200
25	SXYQ1927-2	2019.06.11	Yangquan City, Shanxi Province, China	*Phlebotomus chinensis*	C_AA050137*	MW192755	MW192751
26	SXYQ1944-1	2019.06.11	Yangquan City, Shanxi Province, China	*Phlebotomus chinensis*	C_AA050140*	MW805220	MW805202
27	SXYQ1944-2	2019.06.11	Yangquan City, Shanxi Province, China	*Phlebotomus chinensis*	MW805191	MW805210	MW805192
28	SXYQ1965-3	2019.06.25	Yangquan City, Shanxi Province, China	*Phlebotomus chinensis*	C_AA050141*	MW805222	MW805204
29	SXYQ1966-2	2019.06.25	Yangquan City, Shanxi Province, China	*Phlebotomus chinensis*	C_AA050142*	MW805223	MW805205
30	SXYQ1966-4	2019.06.25	Yangquan City, Shanxi Province, China	*Phlebotomus chinensis*	C_AA050143*	MW805224	MW805206
31	SXYQ1966-5	2019.06.25	Yangquan City, Shanxi Province, China	*Phlebotomus chinensis*	C_AA050144*	MW805225	MW805207
32	SXYQ1968-2	2019.06.25	Yangquan City, Shanxi Province, China	*Phlebotomus chinensis*	C_AA050146*	MW805226	MW805208
33	SXYQ1968-3	2019.06.25	Yangquan City, Shanxi Province, China	*Phlebotomus chinensis*	C_AA050147*	MW805227	MW805209

Note: '/' indicates no reference in database, * sequences obtained in this study.
