# Supplementary material for: Genetic Characteristics of Wuxiang Virus in Shanxi Province, China
Source: Viruses. 2024 Jan 10;16(1):103. doi: 10.3390/v16010103 (PMC10818450; doi:10.3390/v16010103)
Supplement: Supplementary file 1 [file viruses-16-00103-s001.zip › Table S2.pdf]

Table S2. The homology analysis of nucleotides and amino acids in ORF sequence in L segment.

No.	Strain	No.																																
		**1**	**2**	**3**	**4**	**5**	**6**	**7**	**8**	**9**	**10**	**11**	**12**	**13**	**14**	**15**	**16**	**17**	**18**	**19**	**20**	**21**	**22**	**23**	**24**	**25**	**26**	**27**	**28**	**29**	**30**	**31**	**32**	**33**
1	SXJX2239	***	98.0	97.5	90.2	90.4	90.2	90.2	90.2	90.4	90.2	90.2	90.3	90.3	90.3	90.2	90.1	90	90.0	90.1	90.1	90.1	89.9	90.1	90.0	90	90.0	90	90.0	90.1	90.2	90.1	90.1	90.1
2	SXJX2241	99.7	***	97.9	90.6	90.6	90.5	90.5	90.6	90.7	90.5	90.6	90.6	90.5	90.6	90.6	90.4	90.4	90.5	90.5	90.5	90.5	90.3	90.5	90.4	90.3	90.4	90.3	90.4	90.5	90.5	90.4	90.4	90.5
3	SXJX2243	99.6	99.6	***	90.2	90.2	90.1	90.1	90.1	90.3	90.1	90.1	90.2	90.2	90.2	90.2	90.0	90.1	90.1	90.1	90.1	90	89.9	90.1	89.9	89.9	89.9	89.9	89.9	90.2	90.2	90.1	90.1	90.2
4	SXWX1903-4	98.7	98.8	98.7	***	97.9	99.1	99.1	97.9	98.0	99.1	97.9	99.3	98.0	98.0	99.8	97.3	97.6	97.6	97.6	97.6	97.4	97.3	97.7	97.2	97.3	97.3	97.3	97.3	97.6	97.8	97.7	97.7	97.6
5	SXWX1913-2	98.8	98.8	98.8	99.5	***	98.0	98.0	98.2	98.3	98.0	98.2	98.2	98.2	98.3	97.9	97.1	97.4	97.5	97.5	97.4	97.3	97.1	97.5	97.1	97.2	97.1	97.1	97.1	97.4	97.6	97.5	97.5	97.4
6	SXWX1913-3	98.8	98.8	98.8	99.6	99.6	***	99.8	98.1	98.2	99.9	98.1	99.4	98.2	98.2	99.2	97.3	97.7	97.8	97.8	97.7	97.4	97.3	97.8	97.3	97.4	97.3	97.3	97.3	97.7	97.9	97.8	97.8	97.7
7	SXWX1917-1	98.8	98.8	98.8	99.6	99.6	99.9	***	98.0	98.1	99.8	98.0	99.3	98.1	98.2	99.1	97.3	97.8	97.8	97.8	97.8	97.4	97.4	97.9	97.3	97.4	97.4	97.3	97.4	97.8	98.0	97.8	97.8	97.8
8	SXWX1917-4	98.8	98.8	98.8	99.5	99.7	99.6	99.6	***	99.6	98.1	99.9	98.2	98.4	99.7	98.0	97.1	97.5	97.5	97.6	97.5	97.3	97.1	97.6	97.1	97.2	97.2	97.1	97.2	97.5	97.7	97.5	97.5	97.5
9	SXWX1918-3	98.7	98.7	98.7	99.4	99.6	99.5	99.5	99.8	***	98.2	99.7	98.3	98.5	99.8	98.1	97.2	97.6	97.6	97.7	97.6	97.4	97.2	97.7	97.2	97.3	97.3	97.2	97.2	97.6	97.8	97.7	97.7	97.6
10	SXWX1920-2	98.7	98.7	98.7	99.5	99.5	99.9	99.9	99.5	99.4	***	98.1	99.3	98.2	98.2	99.1	97.3	97.7	97.7	97.8	97.7	97.4	97.3	97.8	97.2	97.3	97.3	97.3	97.3	97.7	97.9	97.8	97.8	97.7
11	SXWX1920-3	98.7	98.7	98.7	99.4	99.6	99.5	99.5	99.9	99.7	99.4	***	98.2	98.5	99.7	98.0	97.1	97.5	97.5	97.6	97.5	97.3	97.1	97.6	97.1	97.2	97.2	97.1	97.2	97.5	97.7	97.5	97.5	97.5
12	SXWX1922-1	98.7	98.7	98.7	99.5	99.5	99.6	99.6	99.5	99.4	99.5	99.4	***	98.3	98.3	99.3	97.4	97.8	97.8	97.9	97.8	97.6	97.5	97.9	97.4	97.5	97.5	97.4	97.5	97.8	98.0	97.9	97.9	97.8
13	SXWX1923-4	98.5	98.5	98.5	99.2	99.4	99.3	99.3	99.6	99.5	99.2	99.5	99.3	***	98.6	98.0	97.1	97.5	97.6	97.6	97.5	97.3	97.1	97.6	97.1	97.2	97.2	97.1	97.2	97.5	97.7	97.5	97.5	97.6
14	SXWX1933	98.8	98.8	98.8	99.5	99.7	99.6	99.6	99.9	99.8	99.5	99.8	99.5	99.6	***	98.1	97.2	97.6	97.7	97.7	97.6	97.4	97.3	97.8	97.2	97.3	97.3	97.2	97.3	97.6	97.8	97.7	97.7	97.6
15	SXWX1813-2	98.7	98.8	98.7	99.8	99.5	99.6	99.6	99.5	99.4	99.5	99.4	99.5	99.3	99.5	***	97.3	97.6	97.7	97.7	97.6	97.4	97.3	97.8	97.3	97.4	97.3	97.3	97.4	97.6	97.8	97.7	97.7	97.6
16	SXYQ1903	98.7	98.7	98.7	99.4	99.5	99.5	99.5	99.6	99.5	99.4	99.5	99.4	99.3	99.6	99.4	***	97.8	97.9	97.9	97.8	99.5	99.6	97.9	99.6	99.6	99.7	99.6	99.6	97.8	98.0	97.9	97.9	97.8
17	SXYQ1916	98.8	98.7	98.8	99.5	99.6	99.5	99.5	99.7	99.6	99.5	99.6	99.5	99.3	99.7	99.5	99.7	***	99.8	99.9	99.8	98.0	97.8	99.2	97.8	97.9	97.9	97.9	97.8	99.1	99.0	98.2	98.2	99.6
18	SXYQ1918-2	98.7	98.7	98.7	99.4	99.5	99.4	99.4	99.6	99.5	99.4	99.5	99.4	99.2	99.6	99.4	99.7	100.0	***	99.9	99.8	98.1	97.9	99.2	97.9	97.9	97.9	98.0	97.9	99.1	99.1	98.2	98.2	99.7
19	SXYQ1919-1	98.8	98.7	98.7	99.4	99.5	99.5	99.5	99.6	99.5	99.4	99.5	99.4	99.3	99.6	99.4	99.7	100.0	100.0	***	99.9	98.1	97.9	99.2	97.9	98.0	98.0	98.0	97.9	99.2	99.1	98.3	98.3	99.7
20	SXYQ1921-3	98.7	98.7	98.7	99.4	99.5	99.4	99.4	99.6	99.5	99.4	99.5	99.4	99.2	99.6	99.4	99.7	100.0	99.9	100.0	***	98.0	97.9	99.2	97.9	97.9	97.9	97.9	97.9	99.1	99.0	98.3	98.3	99.7
21	SXYQ1931	98.7	98.7	98.7	99.4	99.5	99.4	99.4	99.5	99.4	99.4	99.4	99.4	99.2	99.5	99.4	99.9	99.7	99.7	99.7	99.7	***	99.5	98.1	99.5	99.6	99.6	99.5	99.5	98.0	98.2	98.1	98.1	98.0
22	SXYQ1941-5	98.6	98.6	98.6	99.3	99.4	99.4	99.4	99.5	99.4	99.3	99.4	99.3	99.2	99.5	99.3	99.9	99.7	99.6	99.7	99.6	99.9	***	98.0	99.6	99.7	99.6	99.6	99.7	97.8	98.1	98.0	98.0	97.9
23	SXYQ1922-2	98.7	98.6	98.6	99.3	99.4	99.4	99.4	99.5	99.4	99.3	99.4	99.3	99.2	99.5	99.3	99.7	99.9	99.8	99.9	99.8	99.7	99.6	***	97.9	98.0	98.0	98.0	98.0	99.2	99.2	98.3	98.3	99.1
24	SXYQ1923-4	98.6	98.6	98.6	99.3	99.4	99.4	99.4	99.5	99.4	99.3	99.4	99.3	99.2	99.5	99.3	99.9	99.7	99.6	99.7	99.6	99.9	99.8	99.6	***	99.6	99.6	99.8	99.6	97.8	98.0	98.0	98.0	97.8
25	SXYQ1927-2	98.7	98.7	98.7	99.4	99.5	99.4	99.4	99.5	99.4	99.4	99.4	99.4	99.2	99.5	99.4	99.9	99.7	99.7	99.7	99.7	99.9	99.9	99.7	99.9	***	99.7	99.6	99.8	97.9	98.1	98.1	98.1	97.9
26	SXYQ1944-1	98.5	98.5	98.5	99.3	99.4	99.3	99.3	99.4	99.3	99.3	99.3	99.3	99.1	99.4	99.3	99.8	99.6	99.5	99.6	99.5	99.8	99.7	99.5	99.7	99.8	***	99.6	99.7	97.9	98.1	98.0	98.0	97.9
27	SXYQ1965-3	98.6	98.6	98.6	99.3	99.4	99.4	99.4	99.5	99.4	99.3	99.4	99.4	99.2	99.5	99.3	99.9	99.7	99.6	99.7	99.6	99.9	99.8	99.6	99.9	99.9	99.7	***	99.6	97.9	98.1	98.0	98.0	97.9
28	SXYQ1966-2	98.6	98.6	98.6	99.3	99.4	99.3	99.3	99.4	99.3	99.3	99.3	99.3	99.1	99.4	99.3	99.8	99.6	99.6	99.6	99.6	99.8	99.8	99.6	99.8	99.8	99.7	99.8	***	97.8	98.1	98.0	98.0	97.8
29	SXYQ1966-4	98.7	98.6	98.6	99.3	99.4	99.4	99.4	99.5	99.4	99.3	99.4	99.3	99.2	99.5	99.3	99.6	99.9	99.8	99.9	99.8	99.6	99.6	99.8	99.6	99.6	99.5	99.6	99.5	***	99.8	98.2	98.2	99.0
30	SXYQ1966-5	98.7	98.7	98.7	99.4	99.5	99.4	99.4	99.5	99.4	99.4	99.4	99.4	99.3	99.5	99.4	99.7	99.9	99.9	99.9	99.9	99.7	99.6	99.8	99.6	99.7	99.5	99.6	99.6	100.0	***	98.4	98.4	99.0
31	SXYQ1968-2	98.6	98.6	98.6	99.3	99.4	99.4	99.4	99.5	99.4	99.3	99.4	99.3	99.2	99.5	99.3	99.7	99.7	99.6	99.7	99.6	99.7	99.6	99.6	99.6	99.7	99.5	99.6	99.6	99.6	99.6	***	100.0	98.2
32	SXYQ1968-3	98.6	98.6	98.6	99.3	99.4	99.4	99.4	99.5	99.4	99.3	99.4	99.3	99.2	99.5	99.3	99.7	99.7	99.6	99.7	99.6	99.7	99.6	99.6	99.6	99.7	99.5	99.6	99.6	99.6	99.6	100.0	***	98.2
33	SXYQ1944-2	98.7	98.6	98.6	99.3	99.4	99.3	99.3	99.5	99.4	99.3	99.4	99.3	99.1	99.5	99.3	99.6	99.9	99.8	99.9	99.8	99.6	99.5	99.7	99.5	99.6	99.4	99.5	99.5	99.7	99.8	99.5	98.6	***

Note: The lower left side of '***' shows the results of amino acid homology, the upper right side of '***' shows the results of nucleotide homology.
