# Supplementary material for: Genetic Characteristics of Wuxiang Virus in Shanxi Province, China
Source: Viruses. 2024 Jan 10;16(1):103. doi: 10.3390/v16010103 (PMC10818450; doi:10.3390/v16010103)
Supplement: Supplementary file 1 [file viruses-16-00103-s001.zip › Table S3.pdf]

Table S3. The homology analysis of nucleotides and amino acids in ORF sequence in M segment.

No.	Strain	No.																																
		**1**	**2**	**3**	**4**	**5**	**6**	**7**	**8**	**9**	**10**	**11**	**12**	**13**	**14**	**15**	**16**	**17**	**18**	**19**	**20**	**21**	**22**	**23**	**24**	**25**	**26**	**27**	**28**	**29**	**30**	**31**	**32**	**33**
1	SXJX2239	***	96.9	91.7	85.2	85.3	85.3	85.4	85.1	85.2	85.3	85.2	85.4	85.3	85.3	85.2	85.3	85.4	85.3	85.4	85.4	85.2	85.5	84.9	85.2	85.1	85.0	85.2	85.2	85.4	85.1	85.6	85.5	85.4
2	SXJX2241	97.0	***	94.1	87.4	87.5	87.5	87.6	87.4	87.4	87.6	87.5	87.6	87.6	87.5	87.5	87.4	87.4	87.4	87.5	87.4	87.3	87.7	86.9	87.3	87.2	87.1	87.3	87.4	87.6	87.3	87.8	87.8	87.5
3	SXJX2243	93.8	96.1	***	87.6	87.6	87.4	87.7	87.7	87.7	87.6	87.7	87.6	87.8	87.8	87.6	87.4	87.4	87.4	87.5	87.5	87.4	87.6	87.0	87.5	87.3	87.2	87.4	87.4	87.5	87.3	87.8	87.7	87.5
4	SXWX1903-4	89.7	92.2	92.3	***	97.0	97.1	97.2	97.1	97.3	97.2	97.2	97.2	97.3	97.3	99.8	96.6	96.7	96.7	96.8	96.7	96.6	96.6	96.5	96.7	96.5	96.4	96.6	96.6	96.6	96.5	96.8	96.9	96.8
5	SXWX1913-2	89.2	91.7	91.7	97.4	***	99.4	99.7	97.2	97.4	99.7	98.8	98.9	97.4	97.5	96.9	96.6	96.7	96.7	96.8	96.8	96.6	96.6	96.6	96.7	96.6	96.5	96.6	96.7	96.5	96.6	96.8	96.9	96.8
6	SXWX1913-3	89.4	91.9	91.9	97.6	99.6	***	99.3	97.2	97.4	99.2	98.6	98.8	97.5	97.5	96.9	96.6	96.7	96.7	96.7	96.7	96.6	96.6	96.6	96.7	96.6	96.6	96.6	96.7	96.5	96.6	96.9	96.9	96.7
7	SXWX1917-1	89.6	92.1	92.1	97.7	99.6	99.6	***	97.4	97.6	99.8	99.0	99.1	97.6	97.7	97.1	96.8	96.9	96.9	97.0	97.0	96.8	96.8	96.7	96.9	96.7	96.7	96.8	96.9	96.7	96.7	97.0	97.0	97.0
8	SXWX1917-4	89.9	92.2	92.4	97.7	97.9	98.3	98.3	***	99.5	97.3	97.2	97.3	98.9	99.6	97.0	96.8	96.8	96.8	96.9	96.8	96.8	96.9	96.6	97.0	96.7	96.6	96.8	96.8	96.7	96.7	97.0	97.0	96.9
9	SXWX1918-3	89.7	92.0	92.1	97.7	97.8	98.2	98.2	99.8	***	97.5	97.4	97.5	99.1	99.8	97.2	97.0	96.9	96.9	97.0	97.0	97.0	96.9	96.7	97.1	96.9	96.8	97.0	97.0	96.7	96.8	97.1	97.1	97.0
10	SXWX1920-2	89.5	92.0	92.1	97.7	99.6	99.5	99.9	98.2	98.1	***	98.9	99.1	97.6	97.6	97.1	96.7	96.9	96.9	96.9	96.9	96.8	96.7	96.7	96.8	96.7	96.6	96.7	96.8	96.6	96.6	96.9	96.9	96.9
11	SXWX1920-3	89.6	92.1	92.2	97.8	99.1	99.2	99.5	98.2	98.0	99.4	***	99.2	97.5	97.5	97.0	96.7	96.9	96.9	96.9	96.9	96.7	96.6	96.7	96.8	96.7	96.6	96.7	96.8	96.6	96.6	96.9	96.9	96.9
12	SXWX1922-1	89.5	92.0	92.0	97.4	98.9	99.0	99.3	97.9	97.8	99.2	99.5	***	97.6	97.6	97.1	96.9	97.0	97.0	97.0	97.0	96.9	96.7	96.8	96.9	96.8	96.7	96.9	96.9	96.7	96.7	97.0	96.9	97.0
13	SXWX1923-4	89.9	92.2	92.4	97.8	97.9	98.2	98.2	99.5	99.4	98.2	98.2	97.9	***	99.2	97.3	97.0	97.2	97.2	97.2	97.2	97.1	97.2	97.0	97.1	96.9	96.8	97.0	97.0	97.0	96.9	97.3	97.3	97.2
14	SXWX1933	89.7	92.1	92.2	97.7	97.9	98.2	98.2	99.9	99.8	98.2	98.1	97.9	99.5	***	97.3	97.1	97.0	97.0	97.1	97.1	97.1	97.0	96.8	97.2	97.0	96.9	97.1	97.1	96.8	96.9	97.2	97.2	97.1
15	SXWX1813-2	89.7	92.1	92.2	99.9	97.3	97.5	97.7	97.7	97.6	97.6	97.7	97.4	97.7	97.7	***	96.5	96.7	96.7	96.7	96.7	96.5	96.5	96.4	96.6	96.4	96.3	96.5	96.5	96.5	96.4	96.8	96.8	96.7
16	SXYQ1903	89.5	91.9	91.9	97.6	97.6	97.9	97.9	98.2	98.2	97.9	98.0	97.9	98.5	98.2	97.5	***	96.9	96.8	96.9	96.8	99.3	96.5	96.7	99.4	99.3	99.2	99.6	99.4	97.1	96.9	96.9	96.8	96.9
17	SXYQ1916	89.7	92.1	91.8	97.7	97.6	97.8	97.9	98.0	97.9	97.9	97.9	97.7	98.2	98.0	97.7	98.2	***	99.7	99.8	99.7	97.0	96.6	98.8	97.0	96.9	96.8	96.9	96.9	98.5	98.6	97.0	97.1	99.7
18	SXYQ1918-2	89.6	92.0	91.7	97.6	97.4	97.7	97.8	97.9	97.8	97.7	97.7	97.6	98.1	97.9	97.5	98.1	99.6	***	99.9	99.7	97.0	96.6	98.8	97.0	96.8	96.8	96.9	96.9	98.5	98.6	97.0	97.1	99.7
19	SXYQ1919-1	89.7	92.1	91.9	97.7	97.6	97.8	97.9	98.0	97.9	97.9	97.9	97.7	98.2	98.0	97.7	98.2	99.8	99.9	***	99.8	97.0	96.7	98.8	97.0	96.9	96.8	96.9	97.0	98.4	98.6	97.1	97.1	99.8
20	SXYQ1921-3	89.7	92.1	91.8	97.7	97.6	97.8	97.9	98.0	97.9	97.9	97.9	97.7	98.2	98.0	97.7	98.2	99.8	99.7	99.9	***	97.0	96.7	98.8	97.0	96.8	96.8	96.9	96.9	98.5	98.7	97.1	97.1	99.7
21	SXYQ1931	89.5	91.9	91.8	97.7	97.6	97.9	97.9	98.2	98.1	97.9	97.9	97.7	98.4	98.2	97.7	99.3	98.4	98.2	98.4	98.4	***	96.6	96.8	99.4	99.3	99.2	99.4	99.4	97.2	97.0	96.9	96.9	97.0
22	SXYQ1941-5	89.9	92.4	92.0	97.7	97.4	97.8	97.8	97.9	97.8	97.7	97.7	97.5	98.1	97.9	97.7	98.1	98.1	97.9	98.1	98.1	98.2	***	96.6	96.6	96.5	96.5	96.6	96.6	96.7	96.6	97.0	97.0	96.7
23	SXYQ1922-2	89.4	91.7	91.3	97.2	97.1	97.4	97.4	97.5	97.4	97.4	97.4	97.2	97.7	97.5	97.1	97.9	99.0	99.0	99.0	99.0	98.0	97.7	***	96.8	96.7	96.6	96.7	96.7	98.3	98.5	96.8	96.9	98.9
24	SXYQ1923-4	89.7	92.1	91.9	98.0	97.8	98.2	98.2	98.4	98.3	98.1	98.1	97.9	98.6	98.4	97.9	99.5	98.7	98.5	98.7	98.7	99.6	98.5	98.3	***	99.5	99.4	99.6	99.6	97.2	97.1	96.8	96.9	97.0
25	SXYQ1927-2	89.7	92.1	91.9	97.8	97.7	98.0	98.0	98.2	98.2	97.9	98.0	97.9	98.5	98.2	97.7	99.4	98.5	98.3	98.5	98.5	99.5	98.3	98.1	99.7	***	99.1	99.4	99.6	97.1	96.9	96.6	96.7	96.9
26	SXYQ1944-1	89.3	91.7	91.5	97.4	97.2	97.6	97.6	97.8	97.7	97.5	97.5	97.4	98.0	97.8	97.4	98.9	98.2	98.0	98.2	98.2	99.0	98.0	97.8	99.3	99.1	***	99.2	99.3	97.0	96.9	96.6	96.6	96.8
27	SXYQ1965-3	89.7	92.1	91.9	97.7	97.6	97.9	97.9	98.2	98.2	97.9	98.0	97.9	98.5	98.2	97.7	99.6	98.4	98.2	98.4	98.4	99.4	98.2	98.0	99.6	99.6	99.0	***	99.5	97.1	97.0	97.0	96.8	96.9
28	SXYQ1966-2	89.7	92.1	91.9	97.8	97.8	98.2	98.2	98.2	98.2	98.1	98.2	98.0	98.5	98.2	97.7	99.4	98.5	98.3	98.5	98.5	99.5	98.3	98.1	99.7	99.7	99.1	99.6	***	97.1	97.0	96.8	96.9	96.9
29	SXYQ1966-4	89.3	91.8	91.4	97.4	97.3	97.7	97.7	97.7	97.7	97.6	97.6	97.4	97.9	97.7	97.4	97.9	99.0	99.2	99.1	99.1	98.1	97.8	98.9	98.4	98.2	97.9	98.1	98.2	***	99.0	96.9	96.9	98.4
30	SXYQ1966-5	89.7	92.1	91.8	97.8	97.7	98.0	98.0	98.1	98.0	97.9	97.9	97.8	98.3	98.1	97.7	98.5	99.3	99.3	99.3	99.3	98.6	98.2	99.1	98.9	98.7	98.4	98.6	98.7	99.5	***	96.8	96.8	98.6
31	SXYQ1968-2	89.9	92.3	92.1	97.4	97.3	97.7	97.5	97.7	97.6	97.4	97.6	97.4	97.9	97.7	97.4	97.9	97.7	97.6	97.7	97.7	97.8	97.9	97.4	98.0	97.9	97.5	98.2	97.9	97.4	97.8	***	99.9	97.1
32	SXYQ1968-3	89.9	92.3	92.1	97.5	97.4	97.7	97.6	97.7	97.6	97.5	97.6	97.4	97.9	97.7	97.4	97.8	97.8	97.7	97.8	97.8	97.9	98.0	97.4	98.1	98.1	97.6	98.1	98.1	97.5	97.9	99.9	***	97.1
33	SXYQ1944-2	89.9	92.3	91.9	97.9	97.7	97.9	98.1	98.2	98.1	98.0	98.0	97.9	98.4	98.2	97.8	98.4	99.8	99.7	99.9	99.9	98.5	98.2	99.2	98.8	98.6	98.3	98.5	98.6	99.3	99.5	97.9	97.9	***

Note: The lower left side of '***' shows the results of amino acid homology, the upper right side of '***' shows the results of nucleotide homology.
