# Supplementary material for: Genetic Characteristics of Wuxiang Virus in Shanxi Province, China
Source: Viruses. 2024 Jan 10;16(1):103. doi: 10.3390/v16010103 (PMC10818450; doi:10.3390/v16010103)
Supplement: Supplementary file 1 [file viruses-16-00103-s001.zip › Table S4.pdf]

Table S4. The homology analysis of nucleotides and amino acids in ORF sequence in NSs gene.

No.	Strain	No.																																
		1	2	3	4	5	6	7	8	9	10	11	12	13	14	15	16	17	18	19	20	21	22	23	24	25	26	27	28	29	30	31	32	33
1	SXJX2239	***	99.6	98.6	88.6	88.3	88.3	88.4	88.8	88.8	88.3	88.2	89.0	88.6	88.8	88.5	89.1	88.8	88.8	88.8	88.9	89.3	89.1	87.6	89.3	89.1	89.4	89.3	89.1	88.0	88.0	88.1	88.1	88.3
2	SXJX2241	99.6	***	98.7	88.8	88.4	88.4	88.5	88.6	88.6	88.4	88.0	89.1	88.8	88.6	88.6	89.3	88.9	88.9	88.9	89.0	89.4	89.3	87.7	89.4	89.3	89.5	89.4	89.3	88.1	88.1	88.3	88.3	88.4
3	SXJX2243	98.5	98.9	***	89.3	88.9	88.9	89.0	89.4	89.4	88.9	88.9	89.7	89.3	89.4	89.1	89.8	89.4	89.4	89.4	89.5	89.9	89.8	88.3	89.9	89.8	90.0	89.9	89.8	88.6	88.6	88.8	88.8	88.9
4	SXWX1903-4	94.3	94.6	95.0	***	98.3	98.3	98.7	97.3	97.6	98.6	97.3	99.1	99.0	97.6	99.6	97.1	96.9	96.9	96.9	97.1	97.1	97.1	96.8	97.2	97.1	97.3	97.2	97.1	96.9	96.9	97.1	97.1	97.1
5	SXWX1913-2	94.3	94.6	94.6	98.1	***	100.0	99.4	97.2	97.4	99.7	97.0	99.0	98.9	97.4	98.5	97.2	96.8	96.8	96.8	96.9	97.2	97.2	96.9	97.3	97.2	97.4	97.3	97.2	97.1	97.1	96.9	96.9	96.9
6	SXWX1913-3	94.3	94.6	94.6	98.1	100.0	***	99.4	97.2	97.4	99.7	97.0	99.0	98.9	97.4	98.5	97.2	96.8	96.8	96.8	96.9	97.2	97.2	96.9	97.3	97.2	97.4	97.3	97.2	97.1	97.1	96.9	96.9	96.9
7	SXWX1917-1	94.6	95.0	95.0	98.5	98.9	98.9	***	97.6	97.8	99.6	97.4	99.4	99.2	97.8	98.9	97.6	97.2	97.2	97.2	97.3	97.3	97.3	97.1	97.4	97.3	97.6	97.4	97.6	97.2	97.2	97.3	97.3	97.3
8	SXWX1917-4	95.0	95.4	95.8	99.2	98.9	98.9	99.2	***	99.7	97.4	100.0	98.0	98.1	99.7	97.4	96.9	96.8	96.8	96.8	96.9	96.9	96.9	96.7	97.1	96.9	97.2	97.1	96.9	96.8	96.8	96.9	96.9	97.2
9	SXWX1918-3	95.0	95.4	95.8	99.2	98.9	98.9	99.2	100.0	***	97.7	99.9	98.2	98.3	100.0	97.7	97.2	97.1	97.1	97.1	97.2	97.2	97.2	96.9	97.3	97.2	97.4	97.3	97.2	97.1	97.1	97.2	97.2	97.4
10	SXWX1920-2	94.6	95.0	95.0	98.5	99.6	99.6	99.2	99.2	99.2	***	97.3	99.2	99.1	97.7	98.7	97.2	97.1	97.1	97.1	97.2	97.2	97.2	97.2	97.3	97.2	97.4	97.3	97.2	97.3	97.3	97.2	97.2	97.2
11	SXWX1920-3	94.4	94.9	95.3	99.6	98.7	98.7	99.1	100.0	100.0	99.1	***	98.0	98.0	99.9	97.4	96.9	96.7	96.7	96.7	96.9	97.0	96.9	96.6	97.0	96.9	97.2	97.0	96.9	96.7	96.7	96.9	96.9	97.2
12	SXWX1922-1	95.0	95.4	95.8	99.2	98.9	98.9	99.2	100.0	100.0	99.2	100.0	***	99.6	98.2	99.2	97.7	97.6	97.6	97.6	97.7	97.7	97.7	97.4	97.8	97.7	98.0	97.8	97.7	97.6	97.6	97.7	97.7	97.7
13	SXWX1923-4	94.6	95.0	95.4	98.9	98.5	98.5	98.9	99.6	99.6	98.9	99.6	99.6	***	98.3	99.1	97.6	97.4	97.4	97.4	97.6	97.6	97.6	97.3	97.7	97.6	97.8	97.7	97.6	97.4	97.4	97.6	97.6	97.6
14	SXWX1933	95.0	95.4	95.8	99.2	98.9	98.9	99.2	100.0	100.0	99.2	100.0	100.0	99.6	***	97.7	97.2	97.1	97.1	97.1	97.2	97.2	97.2	96.9	97.3	97.2	97.4	97.3	97.2	97.1	97.1	97.2	97.2	97.4
15	SXWX1813-2	94.3	94.6	95.0	99.2	98.1	98.1	98.5	99.2	99.2	98.5	99.6	99.2	98.9	99.2	***	97.2	97.1	97.1	97.1	97.2	97.2	97.2	96.9	97.3	97.2	97.4	97.3	97.2	97.1	97.1	97.2	97.2	97.2
16	SXYQ1903	93.9	94.3	94.6	98.1	97.7	97.7	98.9	98.9	98.9	98.1	99.1	98.9	98.5	98.9	98.1	***	98.3	98.3	98.3	98.5	99.5	99.5	96.9	99.6	99.5	99.7	99.6	99.7	97.2	97.2	97.2	97.2	98.2
17	SXYQ1916	94.6	95.0	95.4	98.1	97.7	97.7	98.1	98.9	98.9	98.1	99.1	98.9	98.5	98.9	98.1	98.5	***	99.7	99.7	99.9	98.3	98.3	96.8	98.5	98.3	98.6	98.5	98.3	96.9	96.9	97.1	97.1	98.3
18	SXYQ1918-2	94.6	95.0	95.4	98.1	97.7	97.7	98.1	98.9	98.9	98.1	99.1	98.9	98.5	98.9	98.1	98.5	99.2	***	99.7	99.9	98.3	98.3	96.8	98.5	98.3	98.6	98.5	98.3	96.9	96.9	97.1	97.1	98.3
19	SXYQ1919-1	95.0	95.4	95.4	98.1	97.7	97.7	98.1	98.9	98.9	98.1	99.1	98.9	98.5	98.9	98.1	98.5	99.2	99.2	***	99.9	98.3	98.3	96.8	98.5	98.3	98.6	98.5	98.3	96.9	96.9	97.1	97.1	98.3
20	SXYQ1921-3	95.0	95.4	95.8	98.5	98.1	98.1	98.5	99.2	99.2	98.5	99.6	99.2	98.9	99.2	98.5	98.9	99.6	99.6	99.6	***	98.5	98.5	96.9	98.6	98.5	98.7	98.6	98.5	97.1	97.1	97.2	97.2	98.5
21	SXYQ1931	94.3	94.6	95.0	98.5	98.1	98.1	98.5	99.2	99.2	98.5	99.6	99.2	98.9	99.2	98.5	98.9	98.9	98.9	98.9	99.2	***	99.5	96.9	99.6	99.5	99.7	99.6	99.5	97.2	97.2	97.2	97.2	98.2
22	SXYQ1941-5	94.3	94.6	95.0	98.5	98.1	98.1	98.5	99.2	99.2	98.5	99.6	99.2	98.9	99.2	98.5	98.9	98.9	98.9	98.9	99.2	99.2	***	96.9	99.9	99.7	99.7	99.9	99.7	97.2	97.2	97.4	97.4	98.2
23	SXYQ1922-2	94.6	95.0	95.4	98.9	98.5	98.5	98.9	99.6	99.6	98.9	100.0	99.6	99.2	99.6	98.9	99.2	99.2	99.2	99.2	99.6	99.6	99.6	***	97.1	96.9	97.2	97.1	96.9	99.1	99.1	98.5	98.5	96.7
24	SXYQ1923-4	94.6	95.0	95.4	98.9	98.5	98.5	98.9	99.6	99.6	98.9	100.0	99.6	99.2	99.6	98.9	99.2	99.2	99.2	99.2	99.6	99.6	99.6	100.0	***	99.9	99.9	100.0	99.9	97.3	97.3	97.6	97.6	98.3
25	SXYQ1927-2	94.6	95.0	95.4	98.9	98.5	98.5	98.9	99.6	99.6	98.9	100.0	99.6	99.2	99.6	98.9	99.2	99.2	99.2	99.2	99.6	99.6	99.6	100.0	100.0	***	99.7	99.9	99.7	97.2	97.2	97.4	97.4	98.2
26	SXYQ1944-1	94.6	95.0	95.4	98.9	98.5	98.5	98.9	99.6	99.6	98.9	100.0	99.6	99.2	99.6	98.9	99.2	99.2	99.2	99.2	99.6	99.6	99.6	100.0	100.0	100.0	***	99.9	99.7	97.4	97.4	97.4	97.4	98.5
27	SXYQ1965-3	94.6	95.0	95.4	98.9	98.5	98.5	98.9	99.6	99.6	98.9	100.0	99.6	99.2	99.6	98.9	99.2	99.2	99.2	99.2	99.6	99.6	99.6	100.0	100.0	100.0	100.0	***	99.9	97.3	97.3	97.6	97.6	98.3
28	SXYQ1966-2	94.3	94.6	95.0	98.5	98.1	98.1	99.2	99.2	99.2	98.5	99.6	99.2	98.9	99.2	98.5	99.6	98.9	98.9	98.9	99.2	99.2	99.2	99.6	99.6	99.6	99.6	99.6	***	97.2	97.2	97.4	97.4	98.2
29	SXYQ1966-4	94.6	95.0	95.4	98.9	98.5	98.5	98.9	99.6	99.6	98.9	100.0	99.6	99.2	99.6	98.9	99.2	99.2	99.2	99.2	99.6	99.6	99.6	100.0	100.0	100.0	100.0	100.0	99.6	***	100.0	98.3	98.3	96.8
30	SXYQ1966-5	94.6	95.0	95.4	98.9	98.5	98.5	98.9	99.6	99.6	98.9	100.0	99.6	99.2	99.6	98.9	99.2	99.2	99.2	99.2	99.6	99.6	99.6	100.0	100.0	100.0	100.0	100.0	99.6	100.0	***	98.3	98.3	96.8
31	SXYQ1968-2	94.6	95.0	95.4	98.9	98.5	98.5	98.9	99.6	99.6	98.9	100.0	99.6	99.2	99.6	98.9	99.2	99.2	99.2	99.2	99.6	99.6	99.6	100.0	100.0	100.0	100.0	100.0	99.6	100.0	100.0	***	100.0	96.9
32	SXYQ1968-3	94.6	95.0	95.4	98.9	98.5	98.5	98.9	99.6	99.6	98.9	100.0	99.6	99.2	99.6	98.9	99.2	99.2	99.2	99.2	99.6	99.6	99.6	100.0	100.0	100.0	100.0	100.0	99.6	100.0	100.0	100.0	***	96.9
33	SXYQ1944-2	93.9	94.3	94.6	98.1	97.7	97.7	98.1	98.9	98.9	98.1	99.1	98.9	98.5	98.9	98.1	98.5	98.5	98.5	98.5	98.9	98.9	98.9	99.2	99.2	99.2	99.2	99.2	98.9	99.2	99.2	99.2	99.2	***

Note: The lower left side of '***' shows the results of amino acid homology, the upper right side of '***' shows the results of nucleotide homology.
