# Supplementary material for: Genetic Characteristics of Wuxiang Virus in Shanxi Province, China
Source: Viruses. 2024 Jan 10;16(1):103. doi: 10.3390/v16010103 (PMC10818450; doi:10.3390/v16010103)
Supplement: Supplementary file 1 [file viruses-16-00103-s001.zip › Table S5.pdf]

Table S5. The homology analysis of nucleotides and amino acids in ORF in N gene.

No.	Strain	No.																																
		**1**	**2**	**3**	**4**	**5**	**6**	**7**	**8**	**9**	**10**	**11**	**12**	**13**	**14**	**15**	**16**	**17**	**18**	**19**	**20**	**21**	**22**	**23**	**24**	**25**	**26**	**27**	**28**	**29**	**30**	**31**	**32**	**33**
1	SXJX2239	***	99.7	98.2	91.5	91.6	91.6	91.5	92.2	92.2	91.6	92.2	91.4	92.2	91.9	91.4	91.8	92.3	92.2	92.2	92.3	91.4	91.9	92	91.9	91.6	91.9	91.9	91.9	92	92	92.3	92	92.3
2	SXJX2241	100	***	98.2	91.5	91.6	91.6	91.5	92.2	92.2	91.6	92.2	91.4	91.9	91.9	91.4	91.8	92.3	92.2	92.2	92.3	91.4	92.2	92	91.9	91.9	91.9	91.9	92.2	91.8	91.8	92.3	92	92.3
3	SXJX2243	100	100	***	90.7	90.8	90.8	90.7	91.5	91.5	90.8	91.5	90.7	91.5	91.2	90.6	91.1	91.9	91.8	91.8	91.9	90.7	91.5	91.5	91.2	91.2	91.2	91.2	91.5	91.5	91.5	91.9	91.6	91.6
4	SXWX1903-4	99.6	99.6	99.6	***	98.9	98.9	98.8	98.2	98.2	98.9	98.2	98.8	99.1	98.2	99.9	98.1	98.1	98.0	98.0	98.1	97.4	98.2	98.1	98.2	98.0	98.2	98.0	98.2	97.8	97.6	97.8	97.8	98.4
5	SXWX1913-2	99.6	99.6	99.6	100.0	***	100.0	99.7	98.5	98.5	99.9	98.5	99.1	99.3	98.5	98.8	98.4	98.4	98.2	98.2	98.4	97.7	98.5	98.4	98.5	98.2	98.5	98.2	98.5	98.1	97.8	98.1	98.1	98.7
6	SXWX1913-3	99.6	99.6	99.6	100.0	100.0	***	99.7	98.5	98.5	99.9	98.5	99.1	99.3	98.5	98.8	98.4	98.4	98.2	98.2	98.4	97.7	98.5	98.4	98.5	98.2	98.5	98.2	98.5	98.1	97.8	98.1	98.1	98.7
7	SXWX1917-1	99.2	99.2	99.2	99.6	99.6	99.6	***	98.4	98.4	99.9	98.4	98.9	99.2	98.4	98.7	98.2	98.2	98.1	98.1	98.2	97.6	98.4	98.2	98.4	98.1	98.4	98.1	98.4	98.0	97.7	98.0	98.0	98.5
8	SXWX1917-4	99.6	99.6	99.6	100.0	100.0	100.0	99.6	***	99.7	98.5	100.0	98.4	98.7	99.7	98.1	98.8	99.1	98.9	98.9	99.1	98.1	98.9	98.8	98.9	98.7	98.9	98.7	98.9	98.5	98.2	98.5	98.5	98.8
9	SXWX1918-3	99.6	99.6	99.6	100.0	100.0	100.0	99.6	100.0	***	98.5	99.7	98.4	98.7	99.7	98.1	98.8	99.1	98.9	98.9	99.1	98.1	98.9	98.8	98.9	98.7	98.9	98.7	98.9	98.5	98.2	98.5	98.5	98.8
10	SXWX1920-2	99.6	99.6	99.6	100.0	100.0	100.0	99.6	100.0	100.0	***	98.5	99.1	99.3	98.5	98.8	98.4	98.4	98.2	98.2	98.4	97.7	98.5	98.4	98.5	98.2	98.5	98.2	98.5	98.1	97.8	98.1	98.1	98.7
11	SXWX1920-3	99.6	99.6	99.6	100.0	100.0	100.0	99.6	100.0	100.0	100.0	***	98.4	98.7	99.7	98.1	98.8	99.1	98.9	98.9	99.1	98.1	98.9	98.8	98.9	98.7	98.9	98.7	98.9	98.5	98.2	98.5	98.5	98.8
12	SXWX1922-1	99.2	99.2	99.2	99.6	99.6	99.6	99.2	99.6	99.6	99.6	99.6	***	99.2	98.4	98.7	98.2	98.2	98.1	98.1	98.2	97.6	98.4	98.2	98.4	98.1	98.4	98.4	98.4	98.0	97.7	98.0	98.0	98.5
13	SXWX1923-4	99.6	99.6	99.6	100.0	100.0	100.0	99.6	100.0	100.0	100.0	100.0	99.6	***	98.7	98.9	98.5	98.5	98.4	98.4	98.5	97.8	98.7	98.5	98.7	98.4	98.7	98.4	98.7	98.5	98.2	98.2	98.2	98.8
14	SXWX1933	99.6	99.6	99.6	100.0	100.0	100.0	99.6	100.0	100.0	100.0	100.0	99.6	100.0	***	98.1	98.8	99.1	98.9	98.9	99.1	98.1	98.9	98.8	98.9	98.7	98.9	98.7	98.9	98.5	98.2	98.5	98.5	98.8
15	SXWX1813-2	99.6	99.6	99.6	100.0	100.0	100.0	99.6	100.0	100.0	100.0	100.0	99.6	100.0	100.0	***	98.2	98.0	97.8	97.8	98.0	97.3	98.1	98.0	98.1	97.8	98.1	97.8	98.1	97.7	97.4	97.7	97.7	98.2
16	SXYQ1903	99.2	99.2	99.2	99.6	99.6	99.6	99.2	99.6	99.6	99.6	99.6	99.2	99.6	99.6	99.6	***	98.9	98.8	98.8	98.9	98.8	99.6	98.9	99.6	99.3	99.6	99.3	99.6	98.7	98.4	98.7	98.7	98.9
17	SXYQ1916	99.6	99.6	99.6	100	100	100	99.6	100	100	100	100	99.6	100	100	100	99.6	***	99.9	99.9	100	98.2	99.1	99.1	99.1	98.8	99.1	98.8	99.1	98.8	98.5	98.9	98.9	98.9
18	SXYQ1918-2	99.2	99.2	99.2	99.6	99.6	99.6	99.2	99.6	99.6	99.6	99.6	99.2	99.6	99.6	99.6	99.2	99.6	***	100	99.9	98.4	98.9	99.2	98.9	98.9	98.9	98.7	98.9	98.7	98.7	98.8	99.1	98.8
19	SXYQ1919-1	99.2	99.2	99.2	99.6	99.6	99.6	99.2	99.6	99.6	99.6	99.6	99.2	99.6	99.6	99.6	99.2	99.6	100	***	99.9	98.4	98.9	99.2	98.9	98.9	98.9	98.7	98.9	98.7	98.7	98.8	99.1	98.8
20	SXYQ1921-3	99.6	99.6	99.6	100	100	100	99.6	100	100	100	100	99.6	100	100	100	99.6	100	99.6	99.6	***	98.2	99.1	99.1	99.1	98.8	99.1	98.8	99.1	98.8	98.5	98.9	98.9	98.9
21	SXYQ1931	98.8	98.8	98.8	99.2	99.2	99.2	98.8	99.2	99.2	99.2	99.2	98.8	99.2	99.2	99.2	98.8	99.2	99.6	99.6	99.2	***	98.9	98.5	99.2	98.9	98.9	98.9	98.9	98	98	98	98.2	98.2
22	SXYQ1941-5	99.6	99.6	99.6	100	100	100	99.6	100	100	100	100	99.6	100	100	100	99.6	100	99.6	99.6	100	99.2	***	99.1	99.7	99.7	99.7	99.5	100	98.8	98.5	98.8	98.8	99.1
23	SXYQ1922-2	99.2	99.2	99.2	99.6	99.6	99.6	99.2	99.6	99.6	99.6	99.6	99.2	99.6	99.6	99.6	99.2	99.6	100	100	99.6	99.6	99.6	***	99.1	99.1	99.1	98.8	99.1	98.9	99.2	99.1	99.1	98.9
24	SXYQ1923-4	99.6	99.6	99.6	100	100	100	99.6	100	100	100	100	99.6	100	100	100	99.6	100	99.6	99.6	100	99.2	100	99.6	***	99.5	99.7	99.7	99.7	98.8	98.5	98.8	98.8	99.1
25	SXYQ1927-2	98.8	98.8	98.8	99.2	99.2	99.2	98.8	99.2	99.2	99.2	99.2	98.8	99.2	99.2	99.2	98.8	99.2	99.6	99.6	99.2	99.2	99.2	99.6	99.2	***	99.5	99.2	99.7	98.5	98.5	98.5	98.8	98.8
26	SXYQ1944-1	99.6	99.6	99.6	100	100	100	99.6	100	100	100	100	99.6	100	100	100	99.6	100	99.6	99.6	100	99.2	100	99.6	100	99.2	***	99.5	99.7	98.8	98.5	98.8	98.8	99.1
27	SXYQ1965-3	99.6	99.6	99.6	100	100	100	99.6	100	100	100	100	99.6	100	100	100	99.6	100	99.6	99.6	100	99.2	100	99.6	100	99.2	100	***	99.5	98.5	98.2	98.5	98.5	98.8
28	SXYQ1966-2	99.6	99.6	99.6	100	100	100	99.6	100	100	100	100	99.6	100	100	100	99.6	100	99.6	99.6	100	99.2	100	99.6	100	99.2	100	100	***	98.8	98.5	98.8	98.8	99.1
29	SXYQ1966-4	99.6	99.6	99.6	100	100	100	99.6	100	100	100	100	99.6	100	100	100	99.6	100	99.6	99.6	100	99.2	100	99.6	100	99.2	100	100	100	***	99.7	98.5	98.5	98.7
30	SXYQ1966-5	99.2	99.2	99.2	99.6	99.6	99.6	99.2	99.6	99.6	99.6	99.6	99.2	99.6	99.6	99.6	99.2	99.6	100	100	99.6	99.6	99.6	100	99.6	99.6	99.6	99.6	99.6	99.6	***	98.5	98.5	98.4
31	SXYQ1968-2	99.6	99.6	99.6	100	100	100	99.6	100	100	100	100	99.6	100	100	100	99.6	100	99.6	99.6	100	99.2	100	99.6	100	99.2	100	100	100	100	99.6	***	99.7	98.7
32	SXYQ1968-3	99.2	99.2	99.2	99.6	99.6	99.6	99.2	99.6	99.6	99.6	99.6	99.2	99.6	99.6	99.6	99.2	99.6	100	100	99.6	99.6	99.6	100	99.6	99.6	99.6	99.6	99.6	99.6	100	99.6	***	98.7
33	SXYQ1944-2	99.6	99.6	99.6	100	100	100	99.6	100	100	100	100	99.6	100	100	100	99.6	100	99.6	99.6	100	99.2	100	99.6	100	99.2	100	100	100	100	99.6	100	99.6	***

Note: The lower left side of '***' shows the results of amino acid homology, the upper right side of '***' shows the results of nucleotide homology.
