# Supplementary material for: Genetic Characteristics of Wuxiang Virus in Shanxi Province, China
Source: Viruses. 2024 Jan 10;16(1):103. doi: 10.3390/v16010103 (PMC10818450; doi:10.3390/v16010103)
Supplement: Supplementary file 1 [file viruses-16-00103-s001.zip › Table S6.pdf]

**Table S6.** The amino acid substitutions in two clades of WUXV.

| Segment (Protein) | Site | Clade 1 (codon) | Clade 2 (codon) |
|---|---|---|---|
| L (RdRp) | 100 | R(AGG) | K(AAG) |
| | 252 | F(TTC) | Y(TAT) |
| | 261 | R(AGG) | K(AAG) |
| | 266 | T(ACA) | S(TCA) |
| | 282 | A(GAT) | V(GTT) |
| | 345 | D(GAT) | E(GAA) |
| | 348 | I(ATC) | V(GTT) |
| | 448 | D(GAC) | E(GAA) |
| | 484 | I(ATC) | V(GTC) |
| | 799 | S(AGT) | I(ATT) |
| | 1653 | I(ATA) | T(ACA) |
| | 1757 | I(ATT) | M(ATG) |
| | 1761 | N(AAC) | S(AGT) |
| | 1780 | I(ATA) | V(GTG) |
| | 1831 | N(AAT) | K(AAA) |
| | 1976 | T(ACC) | A(GCC) |
| | 2073 | V(GTT) | I(ATT) |
| M (Gp) | 2 | F(TTT) | I(ATT) |
| | 93 | T(ACA) | S(TCA) |
| | 101 | R(AGG) | K(AAG) |
| | 105 | H(CAT) | Y(TAT) |
| | 142 | A(GCT); T(ACT) | D(GAT); N(AAT) |
| | 143 | A(GCT) | V(GTT) |
| | 145 | D(GAT) | E(GAG) |
| | 210 | N(AAT) | S(AGC) |
| | 218 | E(GAA) | G(GGG) |
| | 219 | K(AAG) | R(AGA) |
| | 237 | E(GAA) | D(GAT) |
| | 255 | S(TCC) | T(ACT) |
| | 271 | E(GAG) | D(GAT) |
| | 281 | S(TCT) | T(ACA) |
| | 284 | S(TCT) | A(GCT) |
| | 289 | P(CCA) | S(TCA) |
| | 292 | V(GTT) | I(ATT) |
| | 297 | I(ATA) | M(ATG) |
| | 306 | L(CTA); I(ATA) | M(ATG) |
| | 312 | A(GCA) | S(TCA) |
| | 313 | S(AGT) | D(GAT) |
| | 314 | Q(CAA) | L(CTA) |

| | | | |
|---|---|---|---|
| | 318 | T(ACA); A(GCA) | K(AAG) |
| | 320 | L(CTG) | P(CCA) |
| | 340 | P(CCC); A(GCC) | D(GAC) |
| | 350 | I(ATA) | V(GTA) |
| | 361 | D(GAC) | S(AGC) |
| | 480 | M(ATG) | L(TTG) |
| | 491 | M(ATG) | V(GTG) |
| | 493 | K(AAG) | Q(CAG) |
| | 496 | S(AGC) | D(GAT) |
| | 514 | D(GAT) | N(AAT) |
| | 530 | E(GAA) | D(GAC) |
| | 623 | M(ATG) | R(AGG) |
| | 627 | Q(CAG) | R(CGG) |
| | 639 | I(ATA) | V(GTG) |
| | 695 | V(GTA) | A(GCA) |
| | 731 | F(TTT) | L(ATT) |
| | 760 | T(ACA); A(GCA) | I(ATA) |
| | 768 | L(CTG) | V(GTG) |
| | 770 | A(GCT) | I(ATC) |
| | 774 | I(ATA) | V(GTA) |
| | 786 | L(TTG) | V(GTG) |
| | 790 | L(CTA) | V(GTC); I(ATC) |
| | 793 | I(ATA) | V(GTA) |
| | 795 | G(GGG) | K(AAG) |
| | 798 | R(AGG) | K(AAG) |
| | 818 | D(GAC) | E(GAA) |
| | 824 | V(GTG) | M(ATG) |
| | 830 | A(GCA) | M(ATG); V(GTT) |
| | 847 | V(GTC) | I(ATC) |
| | 1032 | S(TCT) | H(CAT) |
| | 1041 | P(CCT) | S(TCT) |
| | 1117 | D(GAC); G(GGC) | N(AAC) |
| | 1230 | T(ACT) | P(CCT) |
| | 1324 | T(ACA) | M(ATG) |
| | 1343 | A(GCT) | I(ATC) |
| | 1346 | I(ATA) | V(GTA) |
| | 1349 | V(GTC) | I(ATC) |
| | 1353 | V(GTC) | I(ATC) |
| S (NSs) | 99 | I(ATC) | N(AAT) |
| | 102 | M(ATG) | L(CTG) |
| | 157 | K(AAG) | R(AGG) |
| | 166 | V(GTT) | A(GCC) |
| | 195 | A(GCA) | T(ACA) |

| | | | |
|---|---|---|---|
| | 206 | P(CCC) | S(TCC) |
| | 212 | H(CAC) | Y(TAT) |
| | 228 | E(GAA) | G(GGG) |
| | 230 | P(CCT) | S(TCT) |
| S (N) | 136 | L(CTT) | I(ATC) |
